# Assessments of mental capacity in psychiatric inpatients: a retrospective cohort study

**DOI:** 10.1186/1471-244X-13-115

**Published:** 2013-04-15

**Authors:** Penelope F Brown, Alex D Tulloch, Charlotte Mackenzie, Gareth S Owen, George Szmukler, Matthew Hotopf

**Affiliations:** 1 Department of Psychological Medicine, Institute of Psychiatry, Weston Education Centre, Cutcombe Road, London SE5 9RJ, UK; 2 Institute of Psychiatry, De Crespigny Park, Box P068, London SE5 8AF, United Kingdom; 3 Kings College London, School of Medicine, London, UK

## Abstract

**Background:**

The Mental Capacity Act 2005 (MCA) was introduced in 2007 to protect vulnerable individuals who lack capacity to make decisions for themselves and to provide a legal framework for professionals to assess incapacity. The impact of the MCA on clinical practice is not known. This study aims to evaluate how frequently mental capacity is assessed in psychiatric inpatients, whether the criteria for determining capacity set out in the MCA are used in practice, and whether this has increased with the introduction of the MCA.

**Method:**

A retrospective cohort study was carried out using a case register of South East London mental health service users. The Case Register Interactive Search (CRIS) system enabled searching and retrieval of anonymised information on patients admitted to the South London and Maudsley NHS Foundation Trust since 2006. The presence and outcomes of documented mental capacity assessments in psychiatric admissions between May 2006 and February 2010 were identified and demographic information on all admissions was retrieved.

**Results:**

Capacity assessments were documented in 1,732/17,744 admissions (9.8%). There was a significant increase in the frequency of capacity assessments carried out over the study period of 0.3 percentage points per month (95% CI 0.26-0.36, p < 0.00001). In only 14.7% of capacity assessments were the MCA criteria for assessing capacity explicitly used.

**Conclusions:**

Over the period of the introduction of the MCA there has been a significant increase in the number of mental capacity assessments carried out on psychiatric inpatients. Although mental health services are considering the issue of capacity more frequently, mental capacity assessments are inconsistently applied and do not make adequate use of MCA criteria.

## Background

The Mental Capacity Act 2005 (MCA) came into force in October 2007 and codifies in statute the common law principles of assessing and managing individuals who lack certain decision-making capacities in England and Wales. It is expected that all doctors and other health professionals are able to assess capacity according to the guidance set out in the MCA, or the Adults with Incapacity (Scotland) Act 2000, and accompanying Codes of Practice. The test for incapacity is laid out in section 2 MCA and involves two stages. Firstly, to lack capacity the individual must be found to suffer from “an impairment of or disturbance in the functioning of the brain or mind”. Secondly, the person must be unable to make the relevant decision at the relevant time because of that impairment, due to lacking one or more of the following abilities: (1) the ability to understand and (2) retain information relevant to the decision; (3) the ability to use or weigh that information as part of the process of making the decision; and (4) the ability to communicate their decision [1].

The introduction of the MCA reflects a general shift in social policy changes and attitudes, especially within healthcare settings, regarding the importance of autonomous decision making and has led to increased interest in how mental capacity is conceptualized and assessed [2-5]. However, there is very little research on how and when capacity assessments are performed in practice, and studies in general medical inpatients suggest capacity assessments are often carried out when the patient is refusing treatment or is difficult to manage [6,7]. Whether there has been a change since the introduction of the MCA is not known. One question that remains unclear despite recent legal reform is how frequently capacity assessments should be performed. A key principle of the MCA is that capacity should be presumed, but when should this presumption be called into question? Incapacity is common in medical inpatients, with over 30% estimated to lack capacity to make treatment decisions, and is often not recognised by clinicians [8]. In psychiatric inpatients the prevalence of incapacity is higher (40-60% [9,10]) and it has been suggested that assessment of capacity should form a core part of inpatient psychiatric assessment [10]. Whether this is being done in practice is not known.

When capacity should be assessed in psychiatric settings is particularly unclear due to the existence of the Mental Health Act 1983 (MHA). While this provides a legal framework for detaining and treating patients for their psychiatric needs without their consent, and without consideration of whether they have capacity or not, the MHA still requires healthcare professionals to determine whether a person has the capacity to consent to or refuse treatment. The MHA Code of Practice (para 23.27-29) states that all assessments of capacity must be recorded in the patients’ notes, and that the capacity of patients with mental disorders can fluctuate and capacity assessments should be made at the time the decision in question needs to be made [11]. Even where treatment under the MHA does not require the patient’s consent, for example medical treatment given to a patient within 3 months of detention under section 3 MHA, it is recommended that the patient’s consent should nevertheless be sought, and their capacity to consent recorded [12] (para 23.37). In addition, for patients detained under the provisions of the MHA, the assessment of capacity is explicitly required in certain specified circumstances, most notably when electro-convulsive therapy (ECT) is offered, or when medication is prescribed after three months. In such instances the medical practitioner must certify in writing that the patient is not capable of understanding the “nature, purpose and likely effects of that treatment” ([13] section 58) and a second opinion must be sought before treatment can be given. In informal psychiatric inpatients (i.e. those not detained under the MHA) an assessment of capacity is fundamental to determining whether the safeguards within the MCA - including the rules on restraint (as laid out in sections 5 and 6 MCA) and the ‘Deprivation of Liberty Safeguards’ (introduced by the Mental Health Act 2007) - apply.

Amid the current complexity in mental health law in England and Wales, the Healthcare Commission (now the Care Quality Commission, or CQC) have recommended routine assessment and recording of mental capacity to consent to treatments and interventions from the start of every informal psychiatric inpatient admission [14]. More recently the CQC has raised concerns that questionable assumptions about patients’ capacity to consent to medication could lead to unlawful treatment in patients detained under the MHA [15]. They recommend good recording of capacity assessments regarding consent to treatment, including a record of the discussions that lie behind the outcome of the assessment [12]. This guidance suggests that, despite the principle of assumed capacity, capacity assessments should be carried out as a matter of routine on all psychiatric inpatients, whether informal or detained under the MHA, but this leads to significant questions about the resources available for such assessments and the subsequent quality of capacity assessments.

There have been no large-scale studies examining the impact of the MCA on clinical practice and the quantity and quality of capacity assessments being carried out. This study aims to evaluate how frequently mental capacity is assessed in psychiatric inpatients, whether the criteria for determining capacity set out in the MCA are used, and whether there has been a change in practice with the introduction of the MCA. We use data from the South London and Maudsley NHS Foundation Trust (SLaM) using its Biomedical Research Centre Case Record Interactive Search, a novel informatics system which allows free text in electronic records to be searched for research purposes [16]. We report the frequency of capacity assessments carried out on psychiatric inpatients and how this has changed over recent years, testing the hypothesis that the implementation of the MCA has led to an increase in the number of capacity assessments being reported. We also examine whether clinicians are using the criteria specified in the MCA for determining incapacity, and the prevalence of incapacity in different patient groups.

## Methods

### Setting and study population

The SLaM BRC Clinical Record Interactive Search (CRIS) provides anonymised, in-depth information derived from electronic medical records relating to secondary mental health care, which includes all specialist care for hospitalization. The protocol for this system has been described elsewhere [16-18]. SLaM provides comprehensive secondary mental health care to a population of approximately 1.3 million residents of four London boroughs (Lambeth, Southwark, Lewisham and Croydon) as well as tertiary care national referral units. Electronic clinical records have been used comprehensively across all SLaM services since 2006 and the CRIS system was developed in 2008 to allow searching and retrieval of anonymised information with over 180,000 cases currently represented on the system. CRIS was approved as a dataset for secondary analysis by Oxfordshire Research Ethics Committee C, reference 08/H0606/71.

### Inclusion criteria

We studied all admissions to SLaM psychiatric wards culminating in a discharge between 1^st^ May 2006 and 31^st^ January 2010, a forty-five month period spanning the introduction of the MCA. These comprised admissions to any inpatient service including older adults, child and adolescent mental health, forensic psychiatry, rehabilitation services and mental health in learning disability in any of the four London boroughs served by SLaM, as well as national specialist referral units (including eating disorders, psychosis and affective disorders units) during the study period were included. Individuals who were admitted on more than one occasion during the study period were counted for each admission, and information was gathered on each admission for the period of the inpatient stay only. Those less than sixteen years of age at the time of their admission were excluded as the MCA is only concerned with individuals aged sixteen years or over.

### Exposure variables

For each admission, we extracted age at admission, gender, ethnicity, diagnosis (based on the 10th edition of the World Health Organization International Classification of Diseases (ICD-10)) and Mental Health Act status of each individual, as well as the type of service or ward they were managed under. When more than one diagnosis was recorded for an individual, the one given closest to date of discharge was used. When an admission had been under a number of sections of the Mental Health Act, the most restrictive section was used (e.g. if a patient had been detained under section 2 and under 3 in the same admission they were recorded as being detained under section 3).

### Capacity assessments

The outcome of interest in this study was the documentation in the notes of a capacity assessment carried out at any time during the admission. The search terms “capacity” and “competence” were initially used to identify documented capacity, however, after preliminary analysis of 100 inpatient records, the term “competence” did not yield any additional assessments to the term “capacity”, and this search term was subsequently dropped. All items of correspondence and progress notes containing the term “capacity” were manually reviewed by PB & CM, and any uses of the term “capacity” which were not related to mental capacity assessments were discarded. For all records of mental capacity assessments, the following information was collected: the decision for which the capacity assessment was carried out (“decision type” e.g. the capacity to consent to medication or the capacity to make a will); whether the capacity assessment was carried out or just suggested by a member of the clinical team and not subsequently done; whether the MCA criteria for assessing capacity were documented; whether a specific document for recording the capacity assessment was used; and whether the patient had or lacked capacity in relation to the decision in question. For patients who had multiple capacity assessments during a single admission, only the first recorded assessment was used in the analysis.

Descriptive analysis of data investigating trends in capacity assessments was carried out using Stata 10. A time trend in the proportion of documented capacity assessments was analysed using linear regression after checking for auto-correlation with the Durbin-Watson test.

## Results

### Main outcome measures

17,744 psychiatric admissions of individuals aged 16 years or over (mean age 41.7, range 16–96 years, s.d 15.5) were identified during the study period (May 2006-February 2010). Table 1 shows the clinical and legal characteristics of the study cohort. The outcome of interest (a documented capacity assessment) was present in 1,732 (9.8%, 95% CI 9.3 - 10.2%) of admissions. In a further 423 (2.4%), a mental capacity assessment was suggested by a member of the clinical team, but no record of the assessment taking place was documented. The majority of capacity assessments were carried out by doctors (1,227/1,732, 70.7%), with the remaining by approved social workers or approved mental health practitioners (308/1,732, 17.8%), nurses (103/1,732, 6.0%), and other members of the multidisciplinary team (34/1,732, 2.0%). In 60 assessments (3.5%) the assessor’s profession was unknown.

**Table 1:** Personal, clinical and legal characteristics of psychiatric admissions during the study period

**Category**	**Number (n = 17,744)**	**(%)**	**Number (%) with documented mental capacity assessment**
**Gender**			
Female	8,147	45.9	824 (10.1)
Male	9,597	54.1	908 (9.5)
**Age Group**			
16-25	2,629	14.8	295 (11.3)
26-35	4,368	24.6	335 (7.7)
36-45	5,184	29.2	376 (7.2)
46-55	2,800	15.7	228 (8.1)
56-65	1,188	6.7	150 (12.7)
66-75	776	4.4	151 (19.3)
76+	799	4.5	197 (24.7)
**Ethnicity**			
White European	10,511	59.2	865 (8.2)
Black African	1,913	10.8	269 (14.1)
Black Caribbean	1,567	8.8	211 (13.5)
Black other	1,946	11.0	212 (10.9)
East Asian	410	2.3	35 (8.5)
South Asian	337	1.9	29 (8.6)
Mixed, other	1,060	6.0	111 (10.5)
**Marital Status**			
Single	11,164	64.6	1,077 (9.4)
Married/civil partnership	2,283	12.9	244 (10.7)
Divorced/separated	2,182	12.3	188 (8.6)
Widowed	658	3.7	141 (21.4)
Not-known	1,157	6.5	82 (7.1)
**Diagnosis**			
Organic and Developmental Disorders	903	5.1	236 (26.0)
Schizophrenia	3,902	22.0	547 (14.0)
Schizoaffective and other Psychotic Disorders	2,102	11.9	268 (12.7)
Bipolar Disorder	1,972	11.1	232 (11.8)
Depression and Neurotic Disorders	3,246	18.3	211 (6.5)
Personality Disorders	909	5.1	55 (6.1)
Substance Misuse Disorders	3,582	20.2	109 (3.0)
Eating Disorders and Other Behavioural Disorders	247	1.4	20 (8.1)
Unknown	881	5.0	54 (6.1)
**Service**			
General Adult Services	11,957	67.4	1,074 (9.0)
PICU	564	3.2	108 (19.0)
MHOA	1,390	7.8	328 (23.7)
CAMHS	255	1.4	28 (11.0)
Specialist	746	4.2	81 (10.9)
Learning Disabilities	60	0.3	24 (40.0)
Addictions	2,527	14.2	16 (0.6)
Forensic	177	1.0	62 (35.0)
Rehab	68	0.4	11 (16.2)
**Mental Health Act Status**			
Informal	10,608	59.8	433 (4.1)
Section 4/5/136	703	34.0	68 (9.8)
Section 2	2,326	13.1	332 (14.3)
Section 3*	3,740	21.1	507 (13.6)
Forensic	367	2.1	92 (25.1)
**Total**	**17,744**	**100**	**1,732 (9.8)**
*Section 3 detained for >3 months	2,201	12.4	353 (22.9)

Capacity assessments were carried out for many different reasons, including capacity to consent to psychiatric admission and treatment, capacity to consent to marriage and divorce, capacity to make a will, capacity to refuse life-saving treatment and capacity to consent to sharing medical records. The numbers of capacity assessments per decision type are summarized in Table 2.

**Table 2:** Frequency of capacity assessments per decision type

**Decision type**	**n**	**%**
Psychiatric admission	752	43.4
Psychiatric treatment including ECT	435	25.1
Aftercare and accommodation	111	6.4
Physical health interventions	174	10.1
Legal issues	59	3.4
Finances, contracts, LPA, AD	75	4.4
Other	126	7.3
**Total**	**1,732**	

(LPA = lasting power of attorney AD = advanced directive).

The criteria for determining mental capacity according to the MCA were reported in 254/1,732 capacity assessments (14.7%). This increased from 11.5% before the implementation of the MCA to 15.5% after. This increase did not reach statistical significance (Chi-squared 3.718, p = 0.052); a similar borderline significant increase was observed using time series analysis (0.13 percentage points per month, 95% CI: -0.007 to 0.268, p = 0.06). In the majority of assessments no criteria, or arbitrary criteria, were reported. In only eight cases (0.5%) was a specific form for documenting mental capacity assessments used.

### Time trend analysis

**Figure 1:**
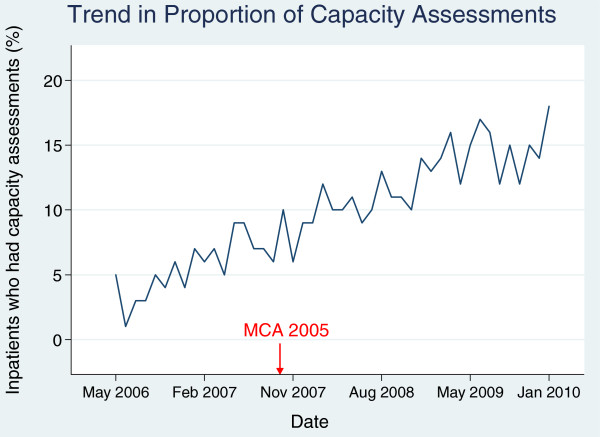
Time trend (per month) of the percentage of inpatients in whom capacity was assessed during their admission.

The frequency of capacity assessments was analysed by month from May 2006 to January 2010. In the first month, 5% of admissions had an assessment; in the final month (January 2010) it was over 17%. Time-series regression indicated a significant increase in the proportion of assessments carried out over the study period (see Figure 1) with no evidence of autocorrelation (Durbin-Watson statistic = 2.22); the regression coefficient was 0.294 (95% CI: 0.258 to 0.328, p < 0.0001), showing a gradual increase in the proportion of approximately 0.3 percentage points per month. There was no step-wise increase in the proportion of inpatients assessed for capacity immediately after the introduction of the MCA in November 2007 (regression coefficient 0.59, 95% CI: -1.21 to 2.39, p = 0.5), perhaps suggesting an anticipatory effect of the change in legislation.

### Outcomes

Overall, 1,101 (63.6%) of the admissions assessed for mental capacity were found to lack capacity. 612 (35.1%) were reported to have capacity, and in 19 (1.1%) of the reported capacity assessments no outcome or an ambiguous outcome (e.g. “fluctuating capacity”) was given. The proportion of admissions lacking capacity varied by diagnosis (see Table 3) with organic and developmental disorders showing the highest prevalence (82.2% and 67.1% respectively). Table 4 shows the proportion of psychiatric admissions found to lack capacity according to MHA status. Patients detained under civil sections of the MHA were more likely to lack capacity than those admitted to hospital voluntarily, however, over 50% of informal patients were found to lack capacity when assessed.

**Table 3:** Proportion of assessments found to lack capacity by diagnosis

**Diagnosis**	**Number (%) of admissions with documented capacity assessment**	**Number assessed to lack capacity**	**% (95% CI) assessed to lack capacity**
Organic and Developmental	236 (26.0)	194	82.2 (77.3-87.1)
Schizophrenia	547 (14.0)	367	67.1 (63.1-71.0)
Schizoaffective/Other Psychotic	268 (12.7)	163	60.8 (54.9-66.7)
Bipolar Disorder	232 (11.8)	160	69.0 (63.0-75.0)
Depression and Neurotic Disorders	211 (6.5)	111	52.6 (45.8-53.4)
Personality Disorders	55 (6.1)	16	29.1 (16.7-41.5)
Substance Misuse Disorders	109 (3.0)	47	43.1 (33.7-52.6)
Eating and other Behavioural Disorders	20 (8.1)	6	30.0 (8.0-52.0)
Unknown	54 (6.1)	37	68.5 (55.7-81.3)
**Total**	**1,732**	**1,101**	**63.6 (61.3-65.8)**

**Table 4:** Proportion of assessments found to lack capacity by MHA status (MHA status at time of assessment)

**MHA status**	**Number (%) of admissions with documented capacity assessment**	**Number (%) assessed to lack capacity**	**% (95% CI) assessed to lack capacity**
**Informal**	637	320	50.3 (46.3-54.1)
**S 4/5/136**	186	149	80.1 (74.3-85.9)
**S2**	324	274	84.6 (80.6-88.5)
**S3**	507	342	67.5 (63.4-71.5)
**Criminal section**	78	16	20.5 (11.3-29.7)
**Total**	**1,732**	**1,101**	**63.6 (61.3-65.8)**

When the proportion of capacity assessments was broken down by month there was a significant decrease in the frequency of incapacity reported over the study period (regression coefficient −0.427, 95% CI −0.623 to −0.230, p = 0.0001) showing a reduction of 0.4 percentage points per month.

## Discussion

For an individual to give their consent to treatment or a procedure they must have the mental capacity to do so, and it is crucial that healthcare professionals are able to assess capacity according to the MCA. Mental capacity should be assessed in relation to specific decisions taken at specific times. This study shows that, in psychiatric clinical practice, capacity is assessed in relation to a variety of decisions, ranging from whether an individual is able to consent to a blood test, to having ECT or to being placed in long-term care homes. We found a significant increase in the proportion of inpatients with documented assessments of capacity in relation to a number of treatment decisions over the period of the implementation of the MCA. This increase was observed both before and after the MCA came into force, suggesting that the legislation itself did not have decisive and immediate effects on practice but provoked anticipatory changes. It is also perhaps associated with a more general shift in culture and increased awareness of capacity issues for psychiatric inpatients. Despite this increase, capacity assessments are infrequently carried out with fewer than 10% of psychiatric inpatients having documented assessments for capacity during their admission.

It is not clear from the legal guidance whether mental capacity should be routinely assessed in all psychiatric inpatients, especially since a key principle of the MCA is that capacity should be presumed. Mental capacity is only of legal relevance in the MHA when obtaining consent to treatment in specific circumstances (ECT and medical treatment after 3 months when the patient is detained under section 3), although it should also be considered when dealing with issues not related to psychiatric treatment such as physical healthcare and welfare issues. Its importance in informal inpatients is less clear and it could thus be argued that assessments of capacity in other circumstances, including informal inpatients, should not be performed routinely unless concern is triggered in some way about the individual’s decision-making ability. On the other hand, previous studies have found that many psychiatric inpatients lack capacity in relation to treatment decisions [9,10,19]. Owen et al. found in the same NHS trust as this study that a quarter of all psychiatric inpatients are both informally admitted and lack capacity relating to treatment decisions [20], raising concerns of deprivations of liberty and best interests decisions for such patients. Should all informal inpatients therefore have a routine capacity assessment? The number of assessments would be substantial and already scarce resources would be put under greater pressure if this were implemented. Perhaps, routine capacity assessments should be restricted to informal patients where there may be a deprivation of liberty? However, it has been shown that lawyers and clinicians are unreliable in assessing deprivation of liberty in informal patients without capacity [21,22], in part because the legal concept of deprivation of liberty is unclear, in contrast to mental capacity which is well-defined and reliably assessed in psychiatric inpatients [4]. The guidance suggests all informal patients should have capacity assessed at the start of admission, whether deprivation of liberty is at issue or not, and it seems hard for clinicians to avoid this obligation to ensure the best interests of their inpatients are served. However, this study shows that capacity assessments were documented in only 433 out of 10, 608 informal admissions (4%), which indicates that mental capacity, let alone deprivation of liberty, is not routinely assessed in practice.

As noted above, one area in which the MHA explicitly requires capacity assessment is in patients who have been receiving medication to treat their mental disorder for three months or more under compulsion. According to section 58 MHA, the responsible clinician must assess the presence of an ability to understand the nature, purpose and likely effects of treatment, and if it is absent, or it is present and the patient is refusing, a second opinion doctor is called upon to certify continued treatment without consent (or treatment against a capable refusal). This safeguard was introduced into the 1983 MHA as a safeguard against heavy drug therapy [23]. In this study capacity assessments were recorded in only 507 out of 3740 admissions under section 3 (14%), and in 23% (353/1539) of those detained for over 3 months. These finding support concerns raised by the Care Quality Commission that many clinicians are not routinely assessing mental capacity and recording their assessments, and failing their statutory requirement in assessing capacity to consent to ongoing treatment under the MHA [24].

The frequency of incapacity, among those tested for it, was approximately 60%. A higher frequency was seen in patients detained under civil sections of the Mental Health Act (67-84%), and a lower frequency in informal patients (50%) consistent with Owen et al’s findings [10]. The frequency of incapacity in patients assessed in forensic services was remarkably lower, with only one in five admissions found to lack capacity. In the entire study population, fewer than a quarter of assessments concerned capacity to consent to treatment, but in forensic wards 87% of assessments were done for this reason. This could be linked to better documentation of capacity assessments as part of consent to treatment provisions under Part IV of the MHA to which the majority of forensic inpatients are subjected. It should be noted that only a minority of inpatients in our sample were assessed for capacity and the true prevalence of incapacity cannot be determined from this study.

Incapacity also varied according to diagnosis, with organic and developmental disorders most commonly leading to incapacity. Incapacity was least common in patients with personality disorders, a group in whom assessment of capacity has been found to be complex [25]. Over the study period the proportion of patients found to lack capacity decreased significantly. It is possible that before the implementation of the MCA, capacity was most often assessed only when it was obviously lacking, hence a high proportion of admissions were found to lack capacity. Over the period of the introduction of the MCA there has been a shift in culture, and capacity is being assessed more routinely and this could explain why fewer individuals who are assessed are now found to lack capacity. However, due to the size of the study it was also not possible to look in depth at the reasons why capacity assessments were being done at particular times and on particular patients.

This is the first large scale study looking at the impact of the MCA on clinical practice. One of its main strengths is the size of the sample, which comprises all admissions one of the largest providers of secondary mental healthcare in Europe. The cohort was limited to a single trust in South London, but this is very large trust covers approximately 2% of the population of England and Wales. We were able to draw on complete electronic clinical records of more than 17,000 inpatients, which provided large amounts of demographic information about each individual, and provided the statistical power to be able to assess the change over time in the assessment of capacity. The disadvantage to using such a large cohort is the lack of qualitative data provided in these results. In order to identify those assessments couched in the appropriate legal terms, capacity assessments were identified only if the term “capacity” was used in the medical records. It is possible that more assessments were carried out but were not documented, or did not use the term “capacity” in the documentation, but due to the large number of patient notes searched in this study it was not feasible to use all potentially relevant search terms, and preliminary searches using the alternative term “competence” yielded no additional documented capacity assessments. It is possible that the use of the term “capacity” also grew during the study period and this could provide further explanation for the rise in documented capacity assessments found in this study.

The assessment of mental capacity can be complex, and the results in this study are limited to what was documented in the electronic notes. The study gives an approximate estimate of when and how capacity was assessed without addressing some of the complexities associated with such assessments. It is likely that capacity assessments are being carried out more frequently and more thoroughly than these results suggest. In a number of cases the records stated the patient lacked capacity without giving reference to an assessment having taken place. The reliance on using the written records makes it difficult to establish whether incapacity was assumed, or whether a full assessment had taken place but was not documented. The electronic records were searched using CRIS, which can access everything in a patient’s medical notes other than scanned documents. This means it was not possible to include hand-written forms for documenting capacity assessments, which were available in SLaM during the study period. The study found that such forms were used in only eight cases, but the actual number is likely to be higher.

This study is limited to the assessment of capacity in psychiatric inpatients and it does not look at the impact of the MCA on practice in non-psychiatric settings. While the rates of incapacity are lower in medical than psychiatric patients [8], there are many instances in general medicine and surgery when an individual’s capacity to consent to care is impaired. Studies have highlighted a lack of training and knowledge amongst psychiatrists [26] and general hospital doctors [27] with regards to the assessment of mental capacity although it has been shown that psychiatrists fare better than general physicians regarding their knowledge of the MCA [28]. It is likely that incapacity is being overlooked more in non-psychiatric settings.

The Healthcare Commission (now the CQC) have recommended routine assessment and recording of mental capacity to consent from the start of every inpatient admission [14], but similar to the findings in this study, they have found that this is not happening in practice [24]. This study also shows that clinicians are rarely using, or rarely document using, the MCA criteria for determining capacity. In fewer than 15% of assessments were the proper criteria for determining mental capacity reported, and in the majority of cases either arbitrary or no criteria were used. While there was a slight improvement in the use of the criteria after the MCA came into force, this suggests there is an ongoing need for training in this area. Studies have shown that capacity can be reliably assessed using a clinical interview and a structured assessment tool [4], while other research has shown that despite the clear guidance in the MCA about how to assess capacity, clinicians have received poor training and lack knowledge of how capacity should be assessed in practice [29,30]. Along with the limited in-depth training in MCA assessments amongst clinicians, the complexities involved in having an older statute (the MHA), where capacity is not of central significance existing alongside the newer MCA, where it is, may explain this current situation. In addition, the wording of the test for incapacity under section 58 MHA (that the patient is “not capable of understanding the nature, purpose and likely effects of that treatment”) differs from the test of capacity set out in the MCA (the patient is unable to understand, retain, or use and weigh information, or cannot communicate their wishes in relation to the decision in question). The existence of two forms of wording for what appears to be the same test is yet another complication that arises from having two statutes.

In this study capacity assessments were predominantly carried out by doctors, and those recorded by approved mental health practitioners mostly concerned the patient’s ability to consent to admission as part of a MHA assessment. However, it is important to note that capacity assessments can be carried out by any health or social care practitioner and indeed both the Social Care Institute for Excellence and the British Psychological Society are developing training materials on mental capacity assessment. Prior to the implementation of the MCA it was observed that professionals varied widely in their understanding of capacity legislation and how and when mental capacity should be assessed [31]. More recently a number of small studies have highlighted ongoing deficiencies in clinicians’ knowledge and use of the MCA [28,30] despite widely available guidance.

## Conclusion

Over the period of the implementation of the MCA there has been a statistically significant increase in the number of mental capacity assessments being recorded in psychiatric inpatients; however, it is clear that the vast majority of patients are still not routinely assessed for mental capacity. This is a potential source of concern in a population where almost two thirds have previously been found to lack capacity to make decisions relating to their treatment or admission, and suggests that in many cases capacity is not being explicitly clarified. This may result in individuals being given medication, subjected to investigations and procedures, or even deprived of their liberty without clear legal protection, both for the individual and the treating clinicians. In addition, despite the MCA introducing a coherent statutory test for determining incapacity, this is only applied in a minority of cases.

While there appears to be a cultural shift throughout psychiatry and healthcare in general to consider mental capacity as a matter of routine, this apparent necessity to demonstrate capacity in all patients undergoing treatment goes against the first principle of the MCA, that a person must be assumed to have capacity unless it is established that he lacks capacity. This paradox needs to be addressed, and currently there is no clear guidance to assist clinicians in deciding when to document that capacity is present, other than in certain instances where the assessment of capacity is obligatory, such as under section 58 of the MHA. In other circumstances capacity assessments are recommended as routine, such as consent to admission for all informal psychiatric inpatients (although it should be noted that capacity and best interests assessments are obligatory when deprivation of liberty is in question). We recommend that further assessment of capacity should be routine if there is a major change in treatment plan or if significant intervention is recommended, and most importantly all assessments should be documented regardless of whether the patient was found to have or lack capacity. This applies not only to psychiatric patients but also to medical patients, and clinicians should be cautious of discriminating against psychiatric patients by presuming incapacity.

There are few interventions in medicine so ethically complex as treating individuals without their consent yet despite increasing access to information and training, clinical practice in assessing capacity remains poor. The MCA Code of Practice provides some guidance, and where there is a reasonable belief that an individual lacks capacity, as is the case for anybody requiring psychiatric inpatient treatment, this should be sufficient to justify carrying out an assessment. But the resource implications of carrying out and documenting detailed assessments of capacity in all psychiatric inpatients in an already understaffed and overstretched health service are not insignificant and need to be considered.

### Unanswered questions and future research

This study raises a number of questions about how, when and why mental capacity assessments are carried out in practice. Are they done due to an increasingly defensive approach to medical practice? Capacity assessments are increasingly becoming part of a routine consent-gaining procedure, especially for complex treatments such as giving ECT, but are they more likely to be done when a patient is refusing rather than accepting the treatment offered to them? This study does not address the motivation behind assessing capacity, and the effects of ethnicity, diagnosis and other clinical factors on whether concerns about incapacity are raised need to be explored further.

## Abbreviations

CQC: Care Quality Commission; CRIS: Case Register Interactive Search; ECT: Electroconvulsive therapy; MCA: Mental Capacity Act 2005; MHA: Mental Health Act 1983 (amended 2007); SLaM: South London and Maudsley NHS Foundation Trust.

## Competing interests

All authors have completed the Unified Competing Interest form at http://www.icmje.org/coi_disclosure.pdf (available on request from the corresponding author) and declare: no support from any organisation for the submitted work.

## Authors’ contribution

PB designed the study and data collection, monitored data collection, carried out statistical analysis, cleaned and analysed the data, and drafted and revised the paper. She is guarantor. AT carried out data collection, cleaned and analysed the data, and drafted and revised the paper. CM collected data and revised the paper. GO and GS revised the draft paper. MH initiated and designed the study, oversaw its conduct, and revised the draft paper. All authors read and approved the final manuscript.

## Pre-publication history

The pre-publication history for this paper can be accessed here:

http://www.biomedcentral.com/1471-244X/13/115/prepub
